# Pod power: Soybean pod and seed photosynthesis contributes to yield

**DOI:** 10.1093/plphys/kiad430

**Published:** 2023-08-01

**Authors:** Alexandra J Burgess, Gustaf E Degen

**Affiliations:** Assistant Features Editor, Plant Physiology, American Society of Plant Biologists; Agriculture and Environmental Sciences, School of Biosciences, University of Nottingham, Sutton Bonington Campus, Loughborough LE12 5RD, UK; Assistant Features Editor, Plant Physiology, American Society of Plant Biologists; Plants, Photosynthesis and Soil, School of Biosciences, University of Sheffield, Firth Court, Western Bank, Sheffield S10 2TN, UK

Photosynthesis uses sunlight energy to assimilate CO_2_ into organic compounds. This remarkable process is the source of most of our food and fuel. Optimizing photosynthesis to adapt crops to climate change and to meet future food demands has resulted in substantial research in this area in recent years. Most research has been focused on photosynthesis in foliar tissue (i.e. leaves), where most of the light harvesting and CO_2_ fixation is catalyzed by the enzyme Rubisco. However, nonfoliar tissues like stem tissue, seeds, or ears of wheat also perform photosynthesis (Schwender et al. 2004; Maydup et al. 2010). Rubisco activity has been detected in nonfoliar tissue of soybean, oilseed rape, and broad bean and in the pod walls of seeds. The Rubisco concentration in these tissues is approximately 10 to 100 times lower than that of leaves (Hedley et al. 1975). Furthermore, nonfoliar tissues have all the required components for light harvesting, such as PSI and PSII, the cytochrome *b_6_f* complex and ATP synthase (Weber et al. 2005; Allorent et al. 2015; Kohzuma et al. 2017). Although both types of tissue fix CO_2_ that has entered the plant through stomata, nonfoliar tissues tend to use a greater amount of CO_2_ released during mitochondrial respiration (Simkin et al. 2020).

A common way to determine the contribution of nonfoliar tissues to photosynthesis is to shade these plant parts (i.e. using aluminium foil) and then measure the effect this has on yield. These types of experiments have shown that wheat ear photosynthesis can contribute up to 70% of individual grain weight yield component in a variety of genotypes, and similar results have been observed for barley (Bort et al. 1994; Maydup et al. 2010). Despite this, more questions remain on how much nonfoliar photosynthesis contributes to yield and seed quality.

In this issue of *Plant Physiology*, Cho et al. (2023) use the light-exclusion method to determine the impact of soybean pod and seed photosynthesis on canopy photosynthesis, seed weight, and seed composition under field conditions (Fig.). Soybean pods were covered in aluminium foil early in development and gas exchange and chlorophyll fluorescence measurements compared with pods and plants that had not been covered. Shading pods resulted in a reduced seed weight of 13% to 14%, similar to previous results on the seed weight of wheat and barley (Maydup et al. 2010; Bort et al. 1994). Furthermore, the operating efficiency of PSII was significantly diminished and covered seeds were visibly much paler due to a decreased chlorophyll content. In contrast to studies on the siliques of Arabidopsis and oilseed rape that identified a reduction in seed oil content (Schwender et al. 2004; Liu et al. 2017), Cho et al. (2023) did not find any significant differences in seed composition of soybean pods dependent on whether they were covered.

**Figure.**
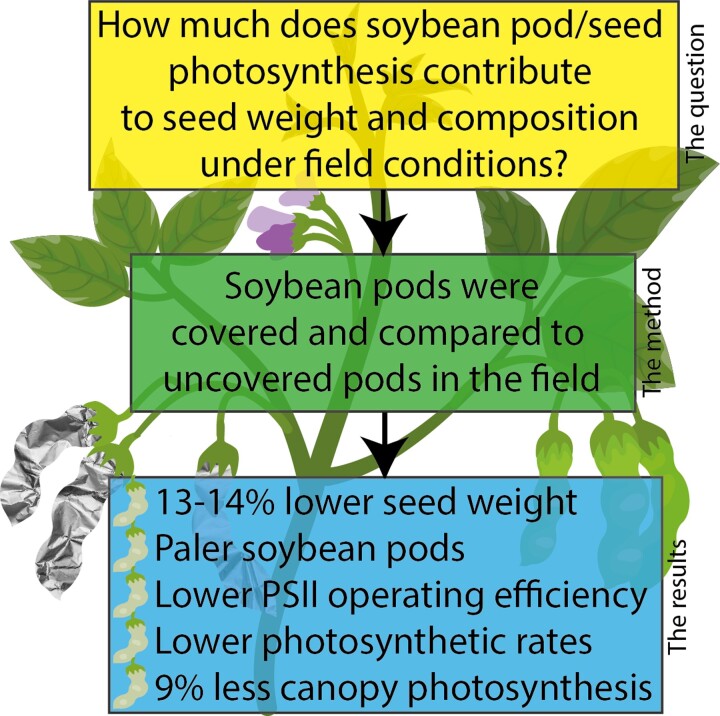
Overview of the impact of pod and seed photosynthesis on soybean yield and photosynthetic productivity following Cho et al. (2023).

Cho et al. (2023) found that both net and gross photosynthesis (the true rate of photosynthesis) were significantly lower in foil-covered soybean pods compared with uncovered pods. Furthermore, uncovered pods had higher rates of dark respiration compared with covered ones. Although uncovered pods were a source of carbon loss for the plant, pod/seed photosynthesis was able to compensate up to 81% of the carbon lost through respiration under field conditions. Using a multilayer canopy model, the authors found that pod/seed photosynthesis contributes up to 9% to total gross canopy photosynthesis. In line with these findings, the authors showed that photosynthesis-related genes were highly expressed in young seeds ahead of peak photosynthetic rates.

All in all, the research by Cho et al. (2023) demonstrates a significant contribution of soybean pod/seed photosynthesis to canopy photosynthesis and yield production, raising the possibility that improving pod/seed photosynthesis can therefore contribute to the global efforts to improve crop productivity. One way this might occur is through optimizing light distribution within the canopy. Soybean pods are located lower down in the canopy and therefore receive relatively low levels of light (<300 *µ*mol m^−2^ s^−1^), well below saturating for these nonfoliar organs. Pod/seed photosynthesis might also play a role in improving nutritional values of soybean pod, with position determining the content of protein and oil in the seed (Huber et al. 2016). Lastly, it would be timely to study the effect of increased atmospheric CO_2_ concentrations and higher temperature on soybean pod photosynthesis using free-air CO_2_ enrichment experiments to gain a better understanding of the effect of anthropogenetic climate change.

## Dive Curated Terms

The following phenotypic, genotypic, and functional terms are of significance to the work described in this paper:

ATP CHEBI: CHEBI:15422
